# mRNA encoding Sec61β, a tail-anchored protein, is localized on the endoplasmic reticulum

**DOI:** 10.1242/jcs.168583

**Published:** 2015-09-15

**Authors:** Xianying A. Cui, Hui Zhang, Lena Ilan, Ai Xin Liu, Iryna Kharchuk, Alexander F. Palazzo

**Affiliations:** University of Toronto, Department of Biochemistry, 1 King's College Circle, MSB Room 5336, Toronto, ON, M5S 1A8, Canada

**Keywords:** Endoplasmic reticulum, mRNA localization, Secretion, Tail-anchored protein

## Abstract

Although one pathway for the post-translational targeting of tail-anchored proteins to the endoplasmic reticulum (ER) has been well defined, it is unclear whether additional pathways exist. Here, we provide evidence that a subset of mRNAs encoding tail-anchored proteins, including Sec61β and nesprin-2, is partially localized to the surface of the ER in mammalian cells. In particular, *Sec61b* mRNA can be targeted to, and later maintained on, the ER using both translation-dependent and -independent mechanisms. Our data suggests that this process is independent of p180 (also known as RRBP1), a known mRNA receptor on the ER, and the transmembrane domain recognition complex (TRC) pathway components, TRC40 (also known as ASNA1) and BAT3 (also known as BAG6). In addition, our data indicates that *Sec61b* mRNA might access translocon-bound ribosomes. Our results show that certain tail-anchored proteins are likely to be synthesized directly on the ER, and this facilitates their membrane insertion. Thus, it is clear that mammalian cells utilize multiple mechanisms to ensure efficient targeting of tail-anchored proteins to the surface of the ER.

## Abstract (precis)

**Highlighted Article:** The mRNA encoding certain tail-anchored proteins is directly localized to the surface of the endoplasmic reticulum, facilitating the insertion of newly synthesized proteins into the membrane.

## INTRODUCTION

One major mechanism that directs proteins to their correct subcellular destination is localization of their mRNA (Holt and Bullock, 2009; Martin and Ephrussi, 2009). Likely the most widespread example is the localization of mRNAs encoding membrane and secreted proteins to the surface of the ER in eukaryotic cells. This localization facilitates the targeting of the encoded proteins to the secretory pathway (Cui and Palazzo, 2014).

Previously it was thought that these mRNAs are exclusively targeted to the ER by their encoded proteins. During their translation, newly synthesized hydrophobic signal sequences or transmembrane domains (TMDs) are recognized as they emerge from the ribosome by the signal recognition particle (SRP), which then redirects the mRNA–ribosome–nascent-chain complex to the ER surface. However, recent studies by our laboratory and other groups demonstrate that a substantial fraction of these mRNAs can be targeted to the ER independently of their translation and the SRP system (Pyhtila et al., 2008; Chen et al., 2011; Cui et al., 2012). This is due in part to the activity of mRNA receptors, such as p180 (also known as RRBP1) (Cui et al., 2012, 2013).

ER localization of mRNAs encoding secretory and membrane-bound proteins might not be universal. Some of these mRNAs appear to be translated by free (i.e. non-ER associated) ribosomes, and their encoded polypeptides are then targeted to the ER post-translationally. One group of membrane proteins thought to be exclusively inserted into membranes post-translationally are tail-anchored proteins (Rabu et al., 2009; Borgese and Fasana, 2011; Hegde and Keenan, 2011). These proteins have a single TMD within the last 50 amino acids from the C-terminus and display their functional N-terminal domain towards the cytosol (Kutay et al., 1993). In mammalian cells, tail-anchored proteins are found on most membranes, including the plasma membrane, ER, Golgi, mitochondria and peroxisomes. In the majority of cases, tail-anchored proteins are first inserted into the ER and then are transported to their proper final destination (Kutay et al., 1995). In addition, it appears that all mitochondrial-targeted and most peroxisome-targeted tail-anchored proteins use specialized pathways. Tail-anchored proteins are involved in many essential cellular processes, such as apoptosis, vesicular transport and protein translocation. Therefore, their correct localization is crucial for cell viability.

For ER-targeted tail-anchored proteins, their targeting is thought to be mediated by the transmembrane domain recognition complex (TRC) pathway. Upon completion of their synthesis, the TMD exits the translating ribosome and is recognized by a series of chaperone proteins which are thought to sort the protein to its proper final destination. These chaperones include SGTA, TRC40 (also known as ASNA1) and BAT3 (also known as BAG6) (Stefanovic and Hegde, 2007; Schuldiner et al., 2008; Jonikas et al., 2009; Leznicki et al., 2010, 2011; Mariappan et al., 2010; Wang et al., 2010). TRC40 then delivers the protein to the ER membrane receptors, WRB and CAML (also known as CAMLG) (Vilardi et al., 2011; Yamamoto and Sakisaka, 2012). Functional orthologs of these proteins in yeast, Get1 and Get2, can mediate membrane insertion (Wang et al., 2014), and the expression of WRB and CAML can complement Get1/2Δ strains (Vilardi et al., 2014). Importantly, this pathway was largely derived from studying the homologous pathway in yeast (the GET pathway) and using mammalian *in vitro* reconstitution assays. However, it remains unclear whether the GET/TRC system is the sole mechanism responsible for targeting tail-anchored proteins to the ER *in vivo*. The idea that there are other pathways is supported by the fact that GET or TRC pathway components can be deleted in yeast (Schuldiner et al., 2005) and mammalian cells (Sasaki et al., 2007) with minimal effects on cell viability, despite the fact that some tail-anchored proteins are necessary for cell homeostasis.

Here, we demonstrate that some mRNAs encoding the tail-anchored proteins, such as nesprin-2 and Sec61β, associate with the ER. Our data suggests that the ER association of *Sec61b* mRNA is not dependent on TRC40, BAT3 or p180. Interestingly, overexpression of *Sec61b* mRNA displaces other mRNAs from the ER, including those that are anchored by translocon-bound ribosomes. This indicates that certain mRNAs encoding tail-anchored proteins can access translocon-bound ribosomes on the surface of the ER and suggests a new alternative pathway for their targeting.

## RESULTS

### *Sec61b* mRNA is partially localized on the ER

It is currently believed that mRNAs encoding tail-anchored proteins are first translated by free ribosomes, and that the encoded polypeptide is later post-translationally targeted to the ER through the TRC pathway (Rabu et al., 2009; Borgese and Fasana, 2011; Hegde and Keenan, 2011).

**Fig. 1.**
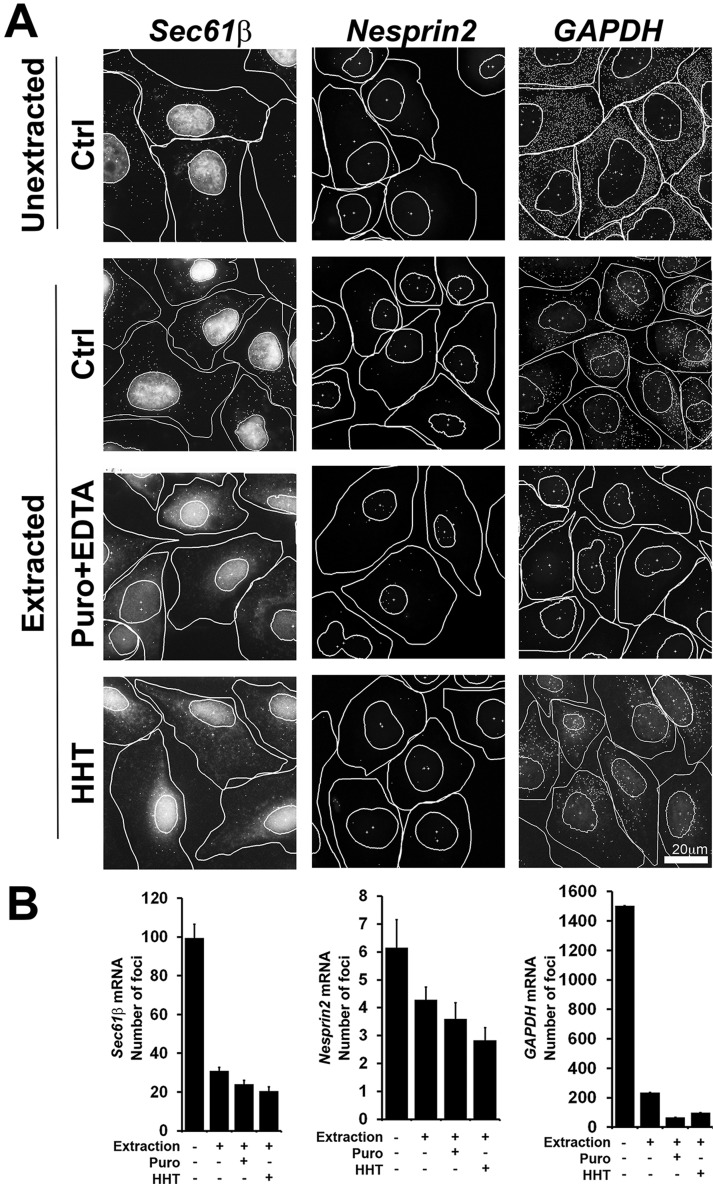
**Endogenous *Sec61b* and nesprin-2 mRNA associates with the ER membrane.** U2OS cells were either: fixed (Unextracted); first extracted with digitonin and then fixed (Extracted); or pre-treated with puromycin (Puro) or homoharringtonine (HHT) for 30 min, extracted with digitonin in the presence or absence of EDTA and then fixed. Cells were stained with a pool of FISH probes to visualize individual endogenous human *Sec61b*, nesprin-2 or *GAPDH* mRNA molecules. Each cell was visualized by phase microscopy to determine the cell contours. mRNA foci were identified using the NIS-element ‘Spot Detection’ function (see Materials and Methods section). (A) mRNA FISH signals overlaid with the contours of the cells and nuclei, and with the detected foci highlighted by the spot detection function. (B) The number of cytoplasmic (i.e. non-nuclear) foci were determined for each condition. Each bar is the mean±s.e.m. of 30 cells. Scale bar: 20 µm.

To assess the distribution of endogenous mRNA in human cells, we stained U2OS cells with a panel of fluorescent *in situ* hybridization (FISH) probes. By simultaneously staining with many probes, one can efficiently visualize individual mRNA molecules (Coassin et al., 2014), as can be seen in Fig. 1. To determine whether these RNAs were tethered to the ER we repeated the experiment in cells that were treated with digitonin, which permeabilizes the plasma membrane and thus extracts the cytosol and removes any molecule that is not associated with the ER (Lerner et al., 2003; Cui et al., 2012; Cui and Palazzo, 2012). By comparing the number of puncta in non-extracted versus extracted cells, we can determine the percentage of mRNAs that are anchored to the ER.

First, we examined the localization of *Sec61b* mRNA*,* which encodes a tail-anchored protein. Sec61β is a component of the translocon, the major protein-conducting channel in the ER, and has been widely used as a model TRC pathway substrate (Borgese and Fasana, 2011). Surprisingly, we found that ∼30% of the endogenous *Sec61b* mRNA was resistant to digitonin extraction (Fig. 1A,B). To test whether the localization of *Sec61b* mRNA was translation dependent, we examined the mRNA localization in cells treated with either homoharringtonine (HHT), or with puromycin followed by extraction with EDTA (Puro+EDTA), two treatments that effectively dissociate ribosomes from mRNA (Cui et al., 2012). To our surprise, most of the ER-localized mRNA was unaffected by these treatments.

Next, we monitored the localization of nesprin-2 (*SYNE2*) mRNA, which encodes a giant tail-anchored protein (796 kDa) that is present on the outer nuclear envelope and is involved in nuclear positioning (Luxton et al., 2010). After extraction, about two thirds of the foci remained, indicating that some of this mRNA was anchored to the ER (Fig. 1A,B). To ensure that the FISH signal was specific, we also probed cells that were depleted of the endogenous nesprin-2 mRNA using RNA interference (RNAi). Indeed, small hairpin RNA (shRNA)-treated cells lost 90% of their signal (supplementary material Fig. S1), indicating that our nesprin-2 probes detected the intended target. Like *Sec61b*, nesprin-2 mRNA largely remained associated with the ER in cells treated with HHT, or puromycin and EDTA. Thus nesprin-2, like *Sec61b*, can associate with the ER membrane, and this activity is mostly independent of translation.

To determine whether partial ER association was a general phenomenon for all mRNAs, we next investigated the localization of an mRNA encoding a cytosolic protein, glyceraldehyde 3-phosphate dehydrogenase (GAPDH). We could reproducibly find 15% of the *GAPDH* puncta in digitonin-extracted cells (Fig. 1A,B). However, in contrast to what we had seen for *Sec61b* and nesprin-2, most of the *GAPDH* mRNAs were extracted in cells treated with either HHT, or puromycin+EDTA (Fig. 1), suggesting that the small amount of ER association was mediated by translating ribosomes.

Thus, we conclude that at least two endogenous mRNAs that encode tail-anchored proteins are also associated with the ER, and this was mostly mediated by contacts that did not involve the ribosome.

### The ORF of *Sec61b* mRNA is required to anchor to the ER independently of translation

**Fig. 2.**
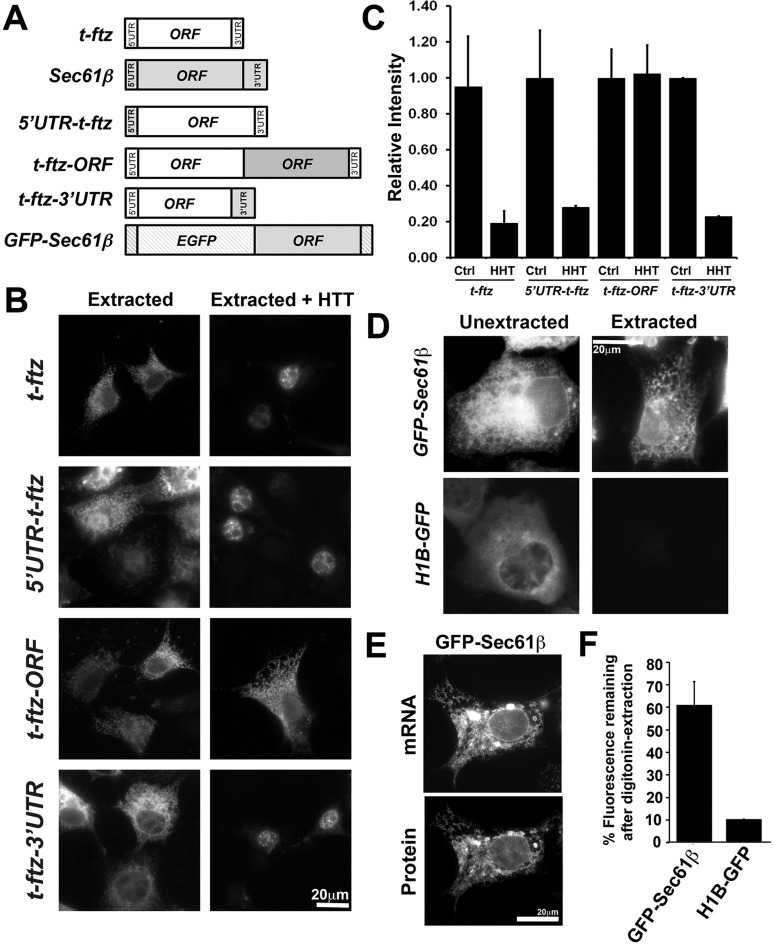
**Overexpressed *GFP-Sec61b* mRNA is associated with the ER membrane.** (A) Schematic diagram of constructs. All *t-ftz* sequences are shown in white, *Sec61b* sequences are shown in gray and *EGFP* sequences are shown as hatched boxes. (B,C) Chimera plasmids containing either the *Sec61b* 5′UTR, 3′UTR or the ORF fused to *t-ftz* were transfected into COS7 cells. At 18–24 h post-transfection, cells were treated with either control or HHT, followed by digitonin extraction to remove cytoplasmic contents. Cells were fixed, stained using FISH probes against *ftz*, and imaged. (D–F) Plasmids encoding *GFP–Sec61b* or *H1B–GFP* were transfected into U2OS cells. At 18–24 h post transfection, cells were either fixed directly (Unextracted) or after digitonin extraction (Extracted). *GFP–Sec61b* or *H1B–GFP* mRNAs were stained with FISH probes against the *GFP*-coding sequence and visualized. mRNAs in unextracted and digitonin-extracted cells are shown in D. Note that *GFP–Sec61b*, but not *H1B–GFP* mRNA, is resistant to digitonin extraction and exhibits a reticular staining pattern. (E) Distribution of GFP–Sec61β protein and mRNA in a digitonin-extracted U2OS cell. Both images are from a single field of view. Note the extensive colocalization of the mRNA with its encoded protein, which is localized to the ER (Rolls et al., 1999; Shibata et al., 2008). (F) Quantification of *GFP–Sec61b* and *H1B–GFP* mRNA cytoplasmic intensity signals. The ratio of fluorescence in the cytoplasms of extracted versus unextracted cells was determined. Each bar in C and F represents the mean±s.e.m. of three independent experiments, each containing at least 30 cells. Scale bars: 20 µm.

We next wanted to identify the region of *Sec61b* mRNA responsible for its ER anchorage. We followed a strategy that we had previously used to identify regions in the placental alkaline phosphatase (*ALPP*) mRNA that promoted ER anchorage (Cui et al., 2013). We fused different regions of *Sec61b* to *t-ftz* (Fig. 2A), an artificial mRNA that encodes a secretory protein and requires translation for ER association (Cui et al., 2012). These constructs were expressed in COS7 cells. After 18–24 h, cells were treated with either control medium or HHT for 30 min to disrupt ribosomes, then extracted to remove non-ER-associated mRNAs, followed by FISH staining to visualize the chimeric mRNAs. To our surprise, versions of *t-ftz* containing either the 5′UTR (*5′UTR-t-ftz*) or 3′UTR (*3′UTR-t-ftz*) of *Sec61b* did not remain anchored to the ER after HHT treatment, resembling the original *t-ftz* mRNA (Fig. 2B, for a quantification of the fluorescence intensity, see Fig. 2C). In contrast, a version of *t-ftz* fused to the *Sec61b* open reading frame (ORF) (*t-ftz-ORF*) remained ER associated after HHT treatment (Fig. 2B). In fact, quantification of the FISH intensities revealed that the level of ER association did not significantly change between control and HHT-treated cells (Fig. 2C).

To further validate these findings, we examined the distribution of *GFP–Sec61b*, a construct that contains the ORF of the human *Sec61b* gene (Fig. 2A). In unextracted COS7 cells, the mRNA had a noticeable reticular-like distribution, suggesting that a large fraction of this mRNA was localized to the ER (Fig. 2D). In digitonin-treated cells, a large portion of the *GFP–Sec61b* mRNA was resistant to extraction (Fig. 2D). In these cells, *GFP–Sec61b* mRNA colocalized with its translated product, GFP–Sec61β protein (Fig. 2E), which is a well-established marker of the ER (Rolls et al., 1999). In contrast, *H1B–GFP* mRNA, which encodes a nuclear histone protein, was mostly extracted by digitonin treatment (Fig. 2D). When the FISH fluorescence levels in extracted and unextracted cells were compared, we observed that 60% of the *GFP–Sec61b* mRNA was resistant to extraction (Fig. 2F). This is comparable to what we have previously observed for other overexpressed mRNAs encoding secreted and membrane-bound proteins (Cui et al., 2012; Cui and Palazzo, 2012). In contrast, only ∼10% of *H1B–GFP* mRNA was resistant to digitonin extraction (Fig. 2F), which is also in line with our previous observations (Cui et al., 2012).

**Fig. 3.**
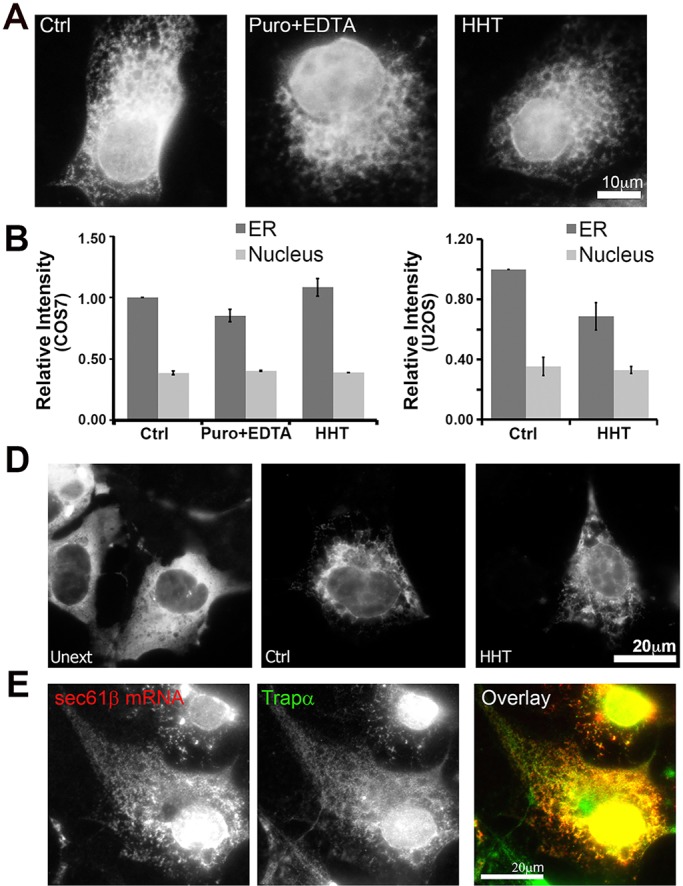
**ER association of overexpressed *GFP–Sec61b* mRNA is partially independent of translation.** (A,B) COS7 and U2OS (C,D) cells were transfected with plasmid encoding *GFP–Sec61b* and allowed to express mRNA for 18–24 h. Cells were then treated with DMSO (Ctrl), puromycin (Puro) or homoharringtonin (HHT) for 30 min, and then extracted with digitonin with or without EDTA. Cells were then fixed and stained for mRNAs using a specific FISH probe against the GFP-coding sequence. Cells were imaged (A,D), and the fluorescent intensities were quantified (B,C). To control for changes in staining, nuclear fluorescent intensities were also analyzed. Each bar represents the mean±s.e.m. of three independent experiments, with each experiment consisting of at least 30 cells. (E) U2OS cells expressing *GFP–Sec61b* were treated with HHT and then digitonin extracted. Cells were then stained for the *GFP–Sec61b* mRNA, and immunostained with the ER marker Trapα. Images in E are from a single field of view including a color overlay showing the *GFP–Sec61b* mRNA in red and Trapα in green. Scale bars: 20 µm.

Next, we assessed whether ER association of *GFP–Sec61b* mRNA required translation. Neither puromycin+EDTA nor HHT treatment disrupted the ER association of *GFP–Sec61b* mRNA in COS7 cells, as assessed by digitonin extraction (Fig. 3A,B). HHT treatment only slightly decreased the ER localization of this mRNA in U2OS cells (Fig. 3C,D). To control for differences in mRNA expression and staining efficiency, we also measured the nuclear fluorescence, and this did not change under any of the tested conditions (Fig. 3B,C). The localization of *GFP–Sec61b* mRNA to the ER in HHT-treated U2OS cells was confirmed by colocalization of the mRNA with the ER marker Trapα (also known as SSR1) (Fig. 3E).

From these experiments, we conclude that the ORF of *Sec61b* mRNA can promote ER association and that this activity is largely independent of its ribosome association and active translation.

### mRNAs encoding other exogenously expressed tail-anchored proteins are mainly localized to the cytosol

To determine whether the results obtained with *GFP–Sec61b* mRNA can be generalized to other mRNAs encoding tail-anchored proteins, we examined the localization of other overexpressed GFP fusion transcripts. In particular, we analyzed the distribution of mRNAs containing ORFs that encode tail-anchored proteins destined to be targeted to the ER (Sec22β and Sec61γ), peroxisome (Pex26) or mitochondria (FIS1). Previously, it has been shown that newly synthesized Pex26 protein is targeted to the peroxisome by Pex19 and thus is independent of the TRC40-dependent pathway (Yagita et al., 2013). For tail-anchored proteins destined for the mitochondria, they are thought to be recognized by a pre-targeting complex which then prevents their sorting to the ER and instead diverts these to the mitochondrial outer membrane (Wang et al., 2010). This sorting process is thought to be dictated by the relative hydrophobicity of the TMD and the presence of charged residues in the vicinity of the TMD (Borgese et al., 2001; Horie, 2003; Wang et al., 2010).

**Fig. 4.**
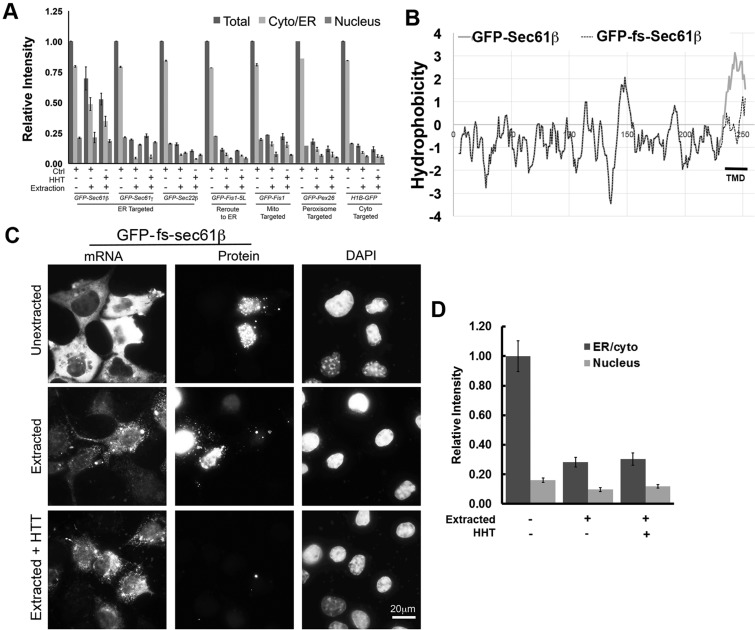
**The coding potential of *GFP-Sec61b* is not required for its localization to the ER.** (A) COS7 cells were transfected with plasmid encoding various GFP-tagged tail-anchored proteins and allowed to express for 18–24 h. The cells were treated with control medium or HHT for 30 min, then either directly fixed or extracted with digitonin and then fixed. Cells were stained for mRNAs using specific FISH probe against the GFP-coding sequence, which was then imaged and quantified. Fluorescent intensities in the cytoplasm and nucleus were quantified. All results were normalized to the cytoplasmic staining intensity in the unextracted cells. Each bar represents the mean±s.e.m. of three independent experiments, each consisting of at least 30 cells. (B) Hydrophobicity (*y*-axis, left) of the polypeptides encoded by *GFP–Sec61b* and *GFP-fs-Sec61b* was plotted against the peptide length (*x*-axis, bottom). Kyte–Doolittle hydropathy values were computed with ProtScale (http://web.expasy.org/protscale/), using a moving window size of 21 amino acids. Note the high hydrophobicity of the TMD region of GFP–Sec61β that is lost in GFP–fs-Sec61β. (C) COS7 cells were transfected with plasmid encoding *GFP–fs-Sec61b* and allowed to express mRNA for 18-–24 h. Cells were then treated with control medium or HHT for 30 min, and then either fixed (Unextracted) or extracted with digitonin and then fixed (Extracted). Cells were stained for mRNAs using a specific FISH probe against the GFP-coding sequence, and for DNA using DAPI. Each row represents a single field of view imaged for *GFP–fs-Sec61b* mRNA, GFP protein and DAPI. (D) Quantification of the cytoplasmic (in unextracted cells), ER (in extracted cells) and nuclear fluorescence intensities of *GFP–fs-Sec61b* mRNA. Each bar represents the mean±s.e.m. of 30 cells. Scale bar: 20 µm.

As expected, GFP–Sec22β and GFP–Sec61γ proteins were targeted to the ER in COS7 cells (data not shown). Likewise, GFP–FIS1 and GFP–Pex26 proteins were targeted, as expected, to the mitochondria (supplementary material Fig. S2A) and peroxisomes (data not shown), respectively. However, unlike *GFP–Sec61b*, all of the other tested mRNAs were efficiently removed by digitonin extraction (Fig. 4A, compare ‘Cyto/ER’ levels in unextracted and extracted cells), similar to what was seen for mRNAs encoding non-secretory proteins (H1B–GFP; Fig. 4A).

We next explored the idea of whether the targeting of a mitochondrial tail-anchored protein to the ER would also increase the amount of ER targeting of its mRNA. When we increased the hydrophobicity of the TMD of FIS1 (FIS1-5L; supplementary material Fig. S2B), the protein was successfully rerouted to the ER (supplementary material Fig. S2A). However, the mRNA of *GFP–FIS1-5L* was still sensitive to extraction and did not localize to the ER (Fig. 4A).

From these experiments, we conclude that ER targeting of the protein product is not sufficient for the ER localization of an mRNA.

### The encoded TMD is not strictly required for the ER localization of *GFP–Sec61b* mRNA

Although ER targeting of the protein product did not correlate with the ER association of the mRNA, it was still possible that *Sec61b* mRNA localization was dependent on its encoded TMD. To further examine this possibility, we frame shifted the TMD of *Sec61b* by inserting a single cytosine nucleotide before the TMD coding region (to create *GFP–fs-Sec61b*). This mutation eliminated the hydrophobic region at the C-terminus of the coding protein (Fig. 4B). Only a few COS7 cells that expressed the *GFP–Sec61b* mRNA showed GFP protein synthesis (for example, see Fig. 4C first row). When it was present, GFP–fs-Sec61β was found in small aggregates that concentrated in the nucleus (see GFP protein localization in Fig. 4C). Consistent with the idea that the translation of the mRNA was not required for ER localization, a fraction of *GFP–fs-Sec61b* mRNA was anchored to the ER (Fig. 4C). When we quantified the amount of mRNA before and after extraction, we found that the amount of ER association in COS7 cells was ∼30% (Fig. 3D), which is about half of what we observed for *GFP–Sec61b* mRNA (see Fig. 2F). This level of ER association was not affected by HHT treatment (Fig. 4D), further confirming that this localization activity occurred independently of ribosomes and translation.

From these results, we conclude that the ER localization of the encoded protein is not required for the localization of *GFP–Sec61b* mRNA. However, given that the targeting of the frame-shifted mutant was clearly decreased from what we had seen with *GFP–Sec61b* mRNA, it is likely that translation of this mRNA into an ER-targeted protein might enhance mRNA localization.

### The initial targeting of *GFP–Sec61b* mRNAs to the ER is partially independent of translation and ribosomes

**Fig. 5.**
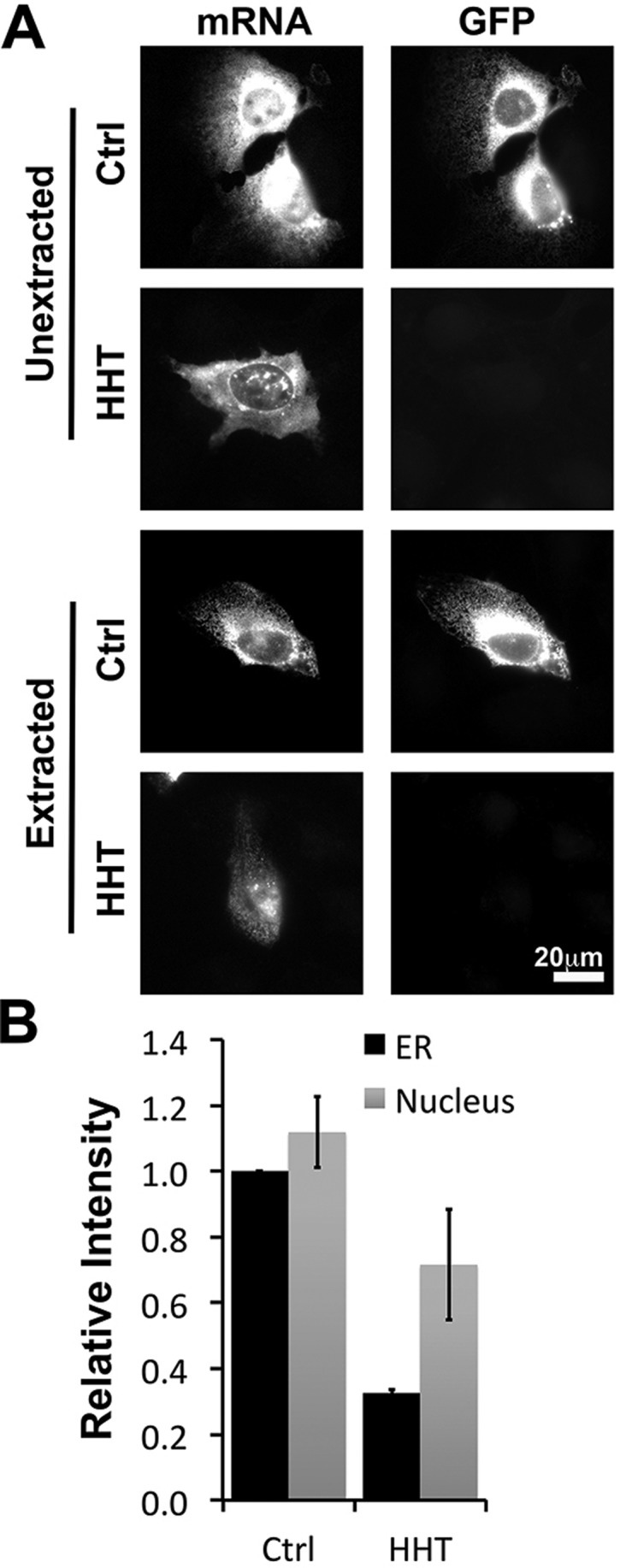
**The initial targeting of *Sec61b* mRNA to the ER is partially dependent on ribosomes and translation.** U2OS cells were pretreated with control medium (Ctrl) or HHT for 15 min, then microinjected with plasmids containing *GFP–Sec61b* and allowed to express mRNAs for 2 h in the presence of medium with or without HHT. The cells were then extracted with digitonin, fixed and stained with FISH probe against the GFP-coding sequence, and imaged. (A) Representative images, with each row representing a single field of view imaged for *GFP–Sec61b* mRNA (mRNA) and GFP fluorescence (GFP). (B) Quantification of the fluorescence intensities of mRNAs in the ER and nucleus of extracted cells. Each bar represents the mean±s.e.m. of three independent experiments, each experiment consisting of at least 30 cells. Scale bar: 20 µm.

Although our data indicated that most ER-targeted *GFP–Sec61b* mRNA could be maintained on the ER independently of translation and ribosomes, we wanted to investigate whether these processes were required for the initial targeting of this mRNA to the membrane. This could potentially explain why more of the *GFP–Sec61b* mRNA was associated with the ER in comparison to *GFP–fs-Sec61b* mRNA. To test this, we microinjected plasmid encoding *GFP–Sec61b* into the nuclei of U2OS cells that were pretreated with either control solution or the translation inhibitor HHT, and examined the targeting of the newly synthesized transcript. As these mRNAs would have never encountered a ribosome, their initial targeting would be strictly mediated by RNA localization pathways. In unextracted cells, mRNAs were efficiently exported out of the nucleus (Fig. 5A). As expected, GFP–Sec61β protein was only made in the control and not the HHT-treated cells, indicating that the translation inhibitor efficiently blocked protein synthesis (Fig. 5A). In extracted cells, *GFP–Sec61b* mRNA was still observed on the ER (Fig. 5A), and by comparing the difference between FISH intensity in unextracted and extracted cells we estimate that ∼70% of the cytosolic mRNA was targeted to the ER. After HHT treatment, ER targeting of the *Sec61b* mRNAs decreased by two thirds (Fig. 5A,B). It is possible that this number underestimates the level of ER targeting, as in the absence of ribosome association, newly synthesized mRNAs might be more efficiently degraded.

In conclusion, these results suggest that although the initial targeting of *Sec61b* mRNA can occur to a certain extent in the absence of translation, it is clearly enhanced in the presence of translating ribosomes.

### p180 is not required for the localization of either *GFP–Sec61b* mRNA or its encoded protein

**Fig. 6.**
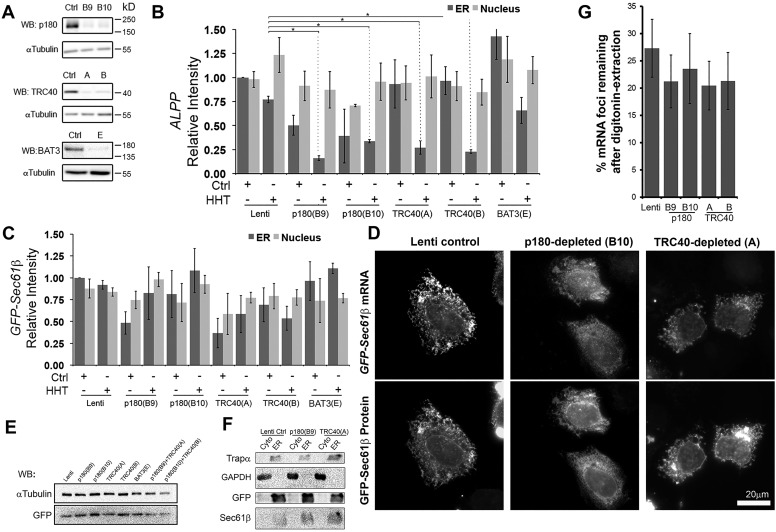
**p180, TRC40 and BAT3 are not required for the ER association of *Sec61b* mRNA and protein.** (A–C) U2OS cells were infected with lentivirus carrying control shRNA (Lenti), or shRNAs against p180 (clones B9 or B10), TRC40 (clones A or B), or BAT3 (clone E). The control and shRNA-infected cells were transfected with plasmids containing either the *ALPP* or *GFP–Sec61b* constructs and allowed to express these mRNAs for 18–24 h. Cells were then treated with either control medium or HHT for 30 min, digitonin extracted, fixed and stained with specific FISH probes, and imaged. (A) Cell lysate was collected on the day of transfection, separated by SDS-PAGE and immunoblotted against p180, TRC40, BAT3 and αtubulin. (B,C) Quantification of the fluorescence intensities of *ALPP* (B) and *GFP–Sec61b* (C) mRNAs, in the ER and nucleus. The results were normalized to the ER staining intensity of cells infected with control shRNA and treated with control medium. Each bar represents the mean±s.e.m. of three or four independent experiments, each experiment consisting of at least 30 cells. *P<0.05 (Student's unpaired *t*-test). (D) shRNA-infected U2OS cells were transfected with plasmids containing *GFP–Sec61b* and allowed to express mRNAs for 18–24 h. The cells were then treated with or HHT for 30 min. Cells were digitonin-extracted, fixed and stained for *GFP–Sec61b* mRNA using FISH probe against GFP-coding region. Each column represents a single field of cells imaged for GFP protein and GFP mRNA. (E,F) shRNA-infected U2OS cells were transfected with plasmids containing *GFP–Sec61b* and allowed to express mRNAs for 18–24 h. Cells were either lysed directly (E) or fractionated into cytosolic (Cyto) and ER fractions (F). The total lysate (E) and fractionated samples (F) were analyzed by immunoblotting using antibody against GFP (GFP–Sec61β), endogenous Sec61β, Trapα (an ER marker) and GAPDH (a cytosolic marker). Depletion of p180 or TRC40 either alone or together did not affect the levels or ER localization of GFP–Sec61β or endogenous Sec61β protein. (G) shRNA-infected U2OS cells that were either digitonin-extracted, or directly fixed were stained with a pool of FISH probes to visualize individual endogenous human *Sec61b* mRNAs. The percentage of cytoplasmic foci remaining in digitonin-extracted versus unextracted cells was calculated. Quantitative results represents the mean±s.e.m. of 30 unextracted and 30 extracted cells. Scale bar: 20 µm.

We previously identified p180 as an mRNA receptor that promoted the ER anchoring of several mRNAs to the ER independently of ribosomes and translation (Cui et al., 2012), and we next tested whether it was required for the localization of *GFP–Sec61b* mRNA. p180 was depleted from U2OS cells using two separate lentiviral-delivered shRNAs (B9 and B10, Fig. 6A). As a positive control, we tested the ER localization of the *ALPP* mRNA. This transcript, which encodes a glycosylphosphatidylinositol (GPI)-anchored protein, can be targeted and maintained on the surface of the ER by the action of p180 (Cui et al., 2012, 2013). Indeed, depletion of p180 with either shRNA construct decreased the ER association of *ALPP* mRNA in both control and HHT-treated cells (Fig. 6B), as we have previously published (Cui et al., 2012). In contrast, depletion of p180 did not consistently decrease the amount of *GFP–Sec61b* mRNA on the ER (Fig. 6C). p180 depletion did not affect ER localization (Fig. 6D) or the overall levels (Fig. 6E) of GFP–Sec61β protein. When various cell fractions were assayed, GFP–Sec61β protein was present in the ER (Fig. 6F), which was consistent with the localization data (Fig. 6D). When we measured the number of individual endogenous *Sec61b* mRNA foci (as in Fig. 1), we observed that p180 depletion did not have a significant impact on the percentage of ER-associated mRNAs (Fig. 6G).

From these observations, we conclude that p180 is dispensable for the ER association of *GFP–Sec61b* mRNA and protein. It formally remains possible that p180 might still play a role, but that other compensatory pathways exist for the ER localization of this mRNA.

### TRC40 and BAT3 are not required for the localization of either *GFP–Sec61b* mRNA or its encoded protein to the ER

As the initial ER targeting of *GFP–Sec61b* mRNA was partially dependent on translation (Fig. 5A,B) and *GFP–fs-Sec61b* mRNA was not as efficiently localized to the ER as *GFP-Sec61b*, it was possible that mRNA localization might be partially coupled to the proper targeting of the encoded protein. In light of this, we assessed whether components of the TRC pathway were required for *GFP–Sec61b* mRNA localization to the ER.

TRC40 and BAT3 were depleted in U2OS cells by lentiviral delivered shRNAs (Fig. 6A), but to our surprise these treatments did not significantly interfere with the ER localization of the GFP–Sec61β protein (for TRC40-depleted cells, see Fig. 6D; for BAT3-depleted cells the data is not shown). Depletion of TRC40 might have had some effect on the amount of ER localization of *GFP–Sec61b* mRNA; however, this varied greatly between experiments (Fig. 6C). TRC40 depletion did not affect GFP–Sec61β protein levels (Fig. 6E) and did not mislocalize the protein to mitochondria (Fig. 6D) or the cytosol (Fig. 6F). Even when both p180 and TRC40 were co-depleted, levels of GFP–Sec61β protein remained constant relative to the loading control (Fig. 6E). Consistent with our observations on overexpressed GFP–Sec61β mRNA and protein, depletion of TRC40 did not have a significant impact on the amount of endogenous *Sec61b* mRNA that was associated to the ER (Fig. 6G).

Unexpectedly, depletion of TRC40 affected the ER localization of *ALPP* mRNA in the HHT-treated cells (Fig. 6B). Interestingly, it has been previously shown that the *S. cerevisiae* ortholog of TRC40, Get3, is required for the ER targeting of GPI-anchored proteins in SRP-disrupted yeast cells (Ast et al., 2013). Our new results suggest that cells depleted of TRC40 might have defects in the localization of certain mRNAs, and this might explain these previous results.

Depletion of BAT3 had no effect on the ER localization of *GFP–Sec61b* and *ALPP* mRNA (Fig. 6B,C).

**Fig. 7.**
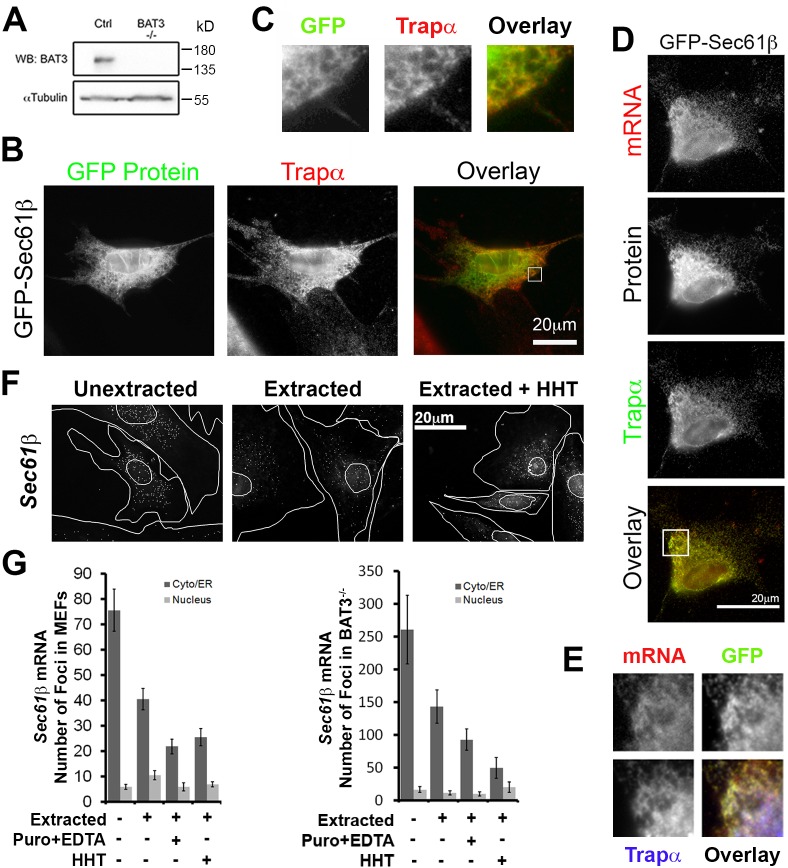
**BAT3 is not required for the ER association of *Sec61b* mRNA and protein.** (A) Western blot of BAT3 protein in control and BAT3^−/−^ MEFs. (B,C) BAT3^−/−^ MEFs expressing *GFP–Sec61b* for 18–24 h were fixed and immunostained for the ER marker Trapα. Images in B are from a single field of view including a color overlay showing the *GFP–Sec61b* mRNA in green and Trapα in red. Higher magnification images of the boxed area in B are shown in C. (D,E) BAT3^−/−^ MEFs expressing *GFP–Sec61b* for 18–24 h were extracted and stained for the *GFP* mRNA by FISH and immunostained for the ER marker Trapα. (D) A single field of view showing *GFP* mRNA, GFP protein, Trapα and an overlay of the *GFP* mRNA (red) and Trapα (green). Higher magnification images of the boxed area are shown in E with an overlay of *GFP–Sec61b* mRNA (red), GFP–Sec61β protein (green) and Trapα (blue). (F) BAT3^−/−^ MEFs were either directly fixed (Unextracted), first extracted with digitonin and then fixed (Extracted), or pre-treated with homoharringtonine (HHT) for 30 min, extracted with digitonin and then fixed. Cells were stained with a pool of FISH probes to visualize individual endogenous mouse *Sec61b* mRNA molecules. (G) The number of cytoplasmic (i.e. non-nuclear) foci were determined for each condition in control MEFs and BAT3^−/−^ cells. Each bar is the mean±s.e.m. of 30 cells. Scale bars: 20 µm.

To confirm the observation that the ER localization of *GFP–Sec61b* mRNA and its encoded protein are mostly independent of the TRC pathway, we repeated these experiments in BAT3-knockout mouse embryonic fibroblasts (MEFs; Fig. 7A). In unextracted cells, exogenously expressed GFP–Sec61β protein clearly colocalized with the ER marker Trapα (Fig. 7B, see high magnification of the boxed area in Fig. 7C), indicating that BAT3 was not required for the ER targeting of this protein. In extracted cells, both the *Sec61b* mRNA and protein colocalized with Trapα (Fig. 7D, high magnification images of the boxed area are shown in Fig. 7E).

We then investigated whether the endogenous *Sec61b* mRNA was associated with the ER. As we had seen previously with U2OS cells, a sizeable number of *Sec61b* mRNA foci were resistant to digitonin extraction in both BAT3^−/−^ cells and wild-type MEFs (∼50%, Fig. 7F,G). The number of foci decreased after ribosomes were disrupted with either HHT or puromycin+EDTA treatments, but was still substantial.

From these results, we conclude that the ER targeting of *GFP–Sec61b* mRNA and its encoded protein was largely independent of the TRC pathway components TRC40 and BAT3. Although it is possible that the small amount of TRC40 remaining after shRNA depletion might be sufficient for the correct targeting of mRNA and/or protein, the fact that these processes are unaltered in BAT3^−/−^ cells suggests that this component is dispensable. Although TRC pathway components might promote mRNA and protein targeting to the ER, our data suggests that other parallel pathways should exist. The presence of these alternative pathways for tail-anchored protein insertion, beyond the TRC pathway, would explain how BAT3-knockout cells are able to survive, despite the fact that certain tail-anchored proteins, such as Sec61γ, are required for cell viability.

### *GFP–Sec61b* mRNA competes with other mRNAs for ribosome binding sites on the ER

In order to understand how different mRNAs associate with the ER and whether they share similar binding sites, we investigated whether two mRNAs could compete with each other (i.e. whether an increase in the levels of one would displace the other from the ER).

We co-expressed *GFP–Sec61b* with two different mRNAs, *t-ftz* and *ALPP*. The first mRNA requires translation for both its targeting and maintenance on the surface of the ER (Cui et al., 2012); thus, we presume that it is anchored to the ER by virtue of the fact that it is being translated by translocon-bound ribosomes. As mentioned above, we have previously demonstrated that >50% of *ALPP* mRNA is associated with the ER in a translation-dependent manner and that the remaining fraction is largely dependent on p180 (Cui et al., 2012).

**Fig. 8.**
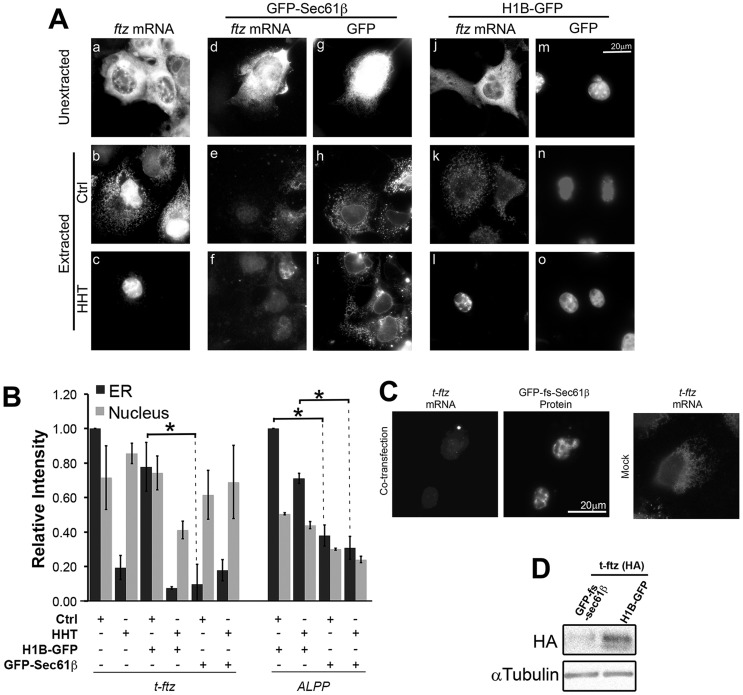
***GFP–Sec61b* mRNA competes with *t-ftz* mRNA for the ribosome-binding sites on the ER.** (A,B) COS7 cells were transfected with plasmid containing a test gene (*t-ftz* or *ALPP*) alone or in combination with plasmid containing a competitor gene (*GFP–Sec61b* or *H1B–GFP*). The cells were then treated with either control medium (Ctrl) or HHT for 30 min, then digitonin extracted, fixed and stained with specific FISH probes, and imaged. (A) Representative images of COS7 cells expressing *t-ftz* mRNA alone (a–c) or in combination with *GFP–Sec61b* (d–i) or *H1B-–GFP* (j–o). Panels a–c are stained for *t-ftz* mRNA, whereas each pair of panels in d–o represents a single field of view imaged for *t-ftz* mRNA and GFP fluorescence. (B) Quantification of the ER and nuclear staining intensity of either *t-ftz* mRNA or *ALPP* mRNA in transfected cells. All data was normalized to the ER staining intensities in the control treated group for each construct. Each bar represents the mean±s.e.m. of three independent experiments, each consisting of at least 30 cells. (C) COS7 cells were transfected with *t-ftz* alone or in combination of *GFP–fs-Sec61b*. At 18–24 h post transfection, cells were digitonin extracted to remove cytoplasmic contents. *GFP–fs-Sec61b* mRNAs were stained with FISH probe against the *GFP*-coding sequence and visualized. (D) Cell lysates of COS7 cells cotransfected with *t-ftz* in combination with either H1B–GFP or GFP–fs-Sec61β were analyzed by western blotting. t-ftz protein expression was examined using HA antibody against an HA epitope present in the t-ftz protein and antibodies against α-tubulin to control for loading. Scale bar: 20 µm.

Interestingly, cells expressing GFP–Sec61β had a significant decrease in the amount of *t-ftz* mRNA on the ER in comparison to cells either expressing *t-ftz* alone or in combination with a control gene (*H1B–GFP*) (Fig. 8A,B). In most cases, no *t-ftz* mRNA could be detected on the ER (Fig. 8A, panel e). In agreement with our previous published results (Cui et al., 2012), *t-ftz* mRNAs were also displaced from the ER upon HHT treatment (Fig. 8A, compare panels c, f and l to panels b, e and k; see Fig. 8B for quantifications), further underscoring the fact that this mRNA requires active translation for ER association. Note that nuclear levels of *t-ftz* remained largely unaltered by GFP–Sec61β expression (see quantification in Fig. 8B).

When *GFP–Sec61b* was co-expressed with *ALPP*, we again observed a decrease in the amount of *ALPP* mRNA on the ER in comparison to cells expressing *ALPP* alone (Fig. 8B). However unlike *t-ftz*, the amount of ER-associated *ALPP* dropped by only 60%. When the co-expressing cells were treated with HHT, the level of *ALPP* mRNA on the ER did not decrease further (Fig. 8B), suggesting the decrease was mainly due to competition between *GFP–Sec61b* and *ALPP* mRNAs for translocon-associated ribosomes.

Thus, it is clear that the expression of *GFP–Sec61b* disrupts the ER localization of other mRNAs. The displacement of *t-ftz* by *GFP-Sec61b* suggests that both of these mRNAs occupy the same ER attachment site, namely translocon-bound ribosomes. It is, however, possible that expression of *GFP–Sec61b* caused some other indirect effects that ultimately resulted in a reduction of mRNA–ER association.

We next tested whether expression of GFP–fs-Sec61β would also displace *t-ftz* mRNA. Unfortunately, many of the cells expressing *GFP–fs-Sec61b* mRNA could not be identified, as few cells express visible levels of protein (for an example, see Fig. 4C). As such, we could not readily identify cells co-expressing both constructs. However, we observed that very few of the cells contained detectable levels of *t-ftz* mRNA in the cytosol after extraction, whether they expressed GFP–fs-Sec61β protein or not (Fig. 8C). If *GFP–fs-Sec61b* mRNA was displacing *t-ftz* mRNA off of the ER, we would also expect that the level of t-ftz protein should decrease in the co-transfected cells. To test this we co-expressed *t-ftz* with either *GFP–fs-Sec61b* or *H1B–GFP*, to control for non-specific competition of translation factors by an overexpressed protein. We found that expression of GFP–fs-Sec61β completely disrupted the production of t-ftz protein (Fig. 8D).

From these results, we conclude that overexpressed *GFP–Sec61b* disrupts the ER localization of other mRNAs and likely perturbs their translation into secretory proteins.

## DISCUSSION

In this paper, we provide evidence that at least one mRNA that encodes a tail-anchored protein is efficiently targeted and then maintained on the surface of the ER. Although our data suggests that mRNA localization does not strictly require active translation and/or ribosomes, it appears that these processes might contribute to the association of this mRNA to the ER.

Overall, our results suggest that multiple pathways exist to target tail-anchored proteins to the ER, including the direct localization of certain mRNAs, such as *Sec61b*, to the surface of the organelle. The encoded protein either might then be spontaneously inserted into the membrane or might use some protein-conducting channel. In agreement with our findings, other groups have also observed that certain mRNAs encoding tail-anchored proteins are associated with the ER (Reid and Nicchitta, 2012; Kraut-Cohen et al., 2013). In particular, Reid and Nichitta observed that 20% of *Sec61b* mRNA is associated with the ER in HEK293 cells (Reid and Nicchitta, 2012), a figure that is close to our measurements (Fig. 1B).

Our data also suggests that once the *GFP–Sec61b* mRNA is at the ER, it might be able to access translocon-bound ribosomes. This finding raises the possibility that the encoded protein of *GFP–Sec61b* mRNA might use translocons to insert into the membrane. Interestingly, the insertion of Sec61β protein into the ER of extracted cells is not affected by translocon depletion (Lang et al., 2012). Moreover, the insertion of this protein, along with most other tail-anchored proteins, into ER-derived rough microsomes requires components of the TRC pathway (Stefanovic and Hegde, 2007). However, these *in vitro* and *ex vivo* results contrast sharply with the *in vivo* situation in which the deletion of components in this pathway is compatible with cellular viability in both yeast (Schuldiner et al., 2005) and mammalian tissue culture cells (Sasaki et al., 2007) despite the fact that many protein substrates are essential. Many of these crucial tail-anchored proteins, such as Sec61γ, whose mRNA does not appear to be ER associated (at least through overexpression), must be able to be correctly inserted into the ER independently of BAT3, as BAT3^−/−^ cells are viable. Intriguingly, newly synthesized Sec61β and other tail-anchored proteins can interact with SRP and translocon components *in vitro* (Abell et al., 2003, 2004). In addition, other HSP40 or HSC70 chaperone systems might also act to promote membrane insertion of these proteins (Rabu et al., 2008). It should also be pointed out that certain tail-anchored proteins, such as cytochrome B5, can spontaneously insert into membranes (Brambillasca et al., 2005; Borgese and Fasana, 2011), and it is possible that localization of the mRNA to the membrane might facilitate this activity. Thus, many pathways likely act redundantly to insert tail-anchored proteins into the ER *in vivo*.

Our final finding that the overexpression of *GFP–Sec61b* displaces other mRNAs off of the ER has one major caveat. Although we have interpreted this observation as being due to the action of the *GFP–Sec61b* mRNA, we cannot totally rule out the possibility that this is due to the expression of the GFP–Sec61β protein. In particular, it is possible that this protein might be incorporated into native Sec61 translocons, which are composed of α, β and γ subunits. These altered translocons would have an additional GFP on their cytosolic face, which would likely prevent the binding of ribosomes and thus impede all translation- or ribosome-dependent anchoring of mRNAs to the ER. We, however, believe that this is unlikely for several reasons. First, GFP–Sec61β protein diffuses in the membrane of the ER at a rate compatible with that of membrane-tethered GFP and not of a large complex such as the translocon (Shibata et al., 2008) (for diffusion measurements of translocons, see Nikonov et al., 2002). Second, translocon disruption is extremely toxic to mammalian tissue culture cells (Lang et al., 2012), whereas the expression of GFP–Sec61β has little to no effect on cell viability. Third, translocons are typically distributed to the perinuclear sheets of the ER and are excluded from both the nuclear envelope and the peripheral tubes (Shibata et al., 2006, 2010), whereas in contrast, GFP–Sec61β is distributed to all three regions (nuclear envelope, sheets and tubes) and does not show a preference for the sheets even when expressed at very low levels (Shibata et al., 2008) (X.A.C. and A.F.P., unpublished observations). It is possible that a minority of translocons incorporate GFP–Sec61β; however, this would not explain why the majority of ER-bound *t-ftz* mRNA would be prevented from accessing translocon-bound ribosomes. Finally, direct perturbation of translocons would not explain why the expression of *GFP–fs-Sec61b* mRNA, which does not encode an ER-targeted protein, also displaces *t-ftz* mRNA from the ER (Fig. 8C).

Finally, it is interesting to note that Sec61β is required for efficient secretion and is an integral part of the endomembrane system. Work from the Nicchitta laboratory has found that mRNAs that encode endomembrane system components have an enhanced, translation-independent affinity for the ER (Chen et al., 2011). Additionally, the association between *Sec61b* mRNA and translocon-bound ribosomes might provide an opportunity for feedback regulation. In this way, translocon availability could potentially be linked to the translation of the *Sec61b* mRNA in order to regulate the production of new translocons and boost secretory capacity.

## MATERIALS AND METHODS

### DNA constructs

The various fragments of the *Sec61b* cDNA were inserted into *t-ftz* pCNDA3 (Palazzo et al., 2007) using restriction-free cloning (van den Ent and Löwe, 2006). For the 5′UTR insertion, the primer pair 5′-CAAGCTTGTCGACGCCGCCACCGCCAGCTGCCGGTCTTTC-3′ and 5′-GGAGCAGCGTGCACGGTACCATATTGGAGATGAGGGTGGCAA-3′ was used. For the ORF insertion, the primer pair 5′-GATGTTCCAGATTACGTCCTGCAGATGCCTGGTCCGACCCCCAG-3′ and 5′-TGGGACAGCAAGAAAGCGAGCTTACGAACGAGTGTACTTGCCCCAAATG-3′ was used. For the 3′UTR insertion, the primer pair 5′-GTTCCAGATTACGTCCTGCAGTAAATTCAGTTACATCCATCTGTCATC-3′ and 5′-AATTGGGACAGCAAGAAAGCGAGCCAGTATAAGTGAATTAAAAAGTTTAT-3′ was used. *FIS1* ORF was amplified from a U2OS cDNA library with forward primer 5′-AGATCTATGGAGGCCGTGCTGAACG-3′ and reverse primer 5′-GAATTCCTTGCTGTGTCCAAGTCCAAATCCTGA-3′. The amplified ORFs were then inserted into the pEGFP-C1 multi-cloning site (MCS) using the EcoRI and BglII sites. To alter the TMD of the FIS1 (GGMALGCAG to LLMALLVLL, see supplementary material Fig. 2B), restriction enzyme-free cloning was performed as previously described (van den Ent and Löwe, 2006) to incorporate five leucine residues into the TMD (forward primer, 5′-TTACTTATGGCCCTGTTGGTGCTTTTGCTGGCCGGACTCATCGGACTTGC-3′ and reverse primer, 5′-CAAAAGCACCAACAGGGCCATAAGTAACACGATGGCCATGCCCACGAGTC-3′). All other genes were amplified from a cDNA library prepared from U2OS cells. For *GFP–Sec22β*, the forward primer 5′-ATGGTGTTGCTAACAATGATCGCC and reverse primer 5′-GTCCGATTCTGGTGGCTGTGA-3′ were used to amplify the *Sec22β* ORF, which was inserted into the TOPO cloning vector (Invitrogen) and subsequently cloned into the pEGFP-C1 vector using the BglII cloning site. For *Sec61γ*, the forward primer 5′-GGCAGAAACCCGGA-3′ and reverse primer- 5′-TTCATTTACTTTGAAATTACTTTAATTTAG-3′ were used to amplify the gene including the UTRs which were subsequently inserted into the MCS of pcDNA3.1 vector. The GFP ORF was then inserted at the N-terminal of the *Sec61γ* sequence using restriction enzyme-free cloning with forward primer, 5′-GGTTGGGTAGGCAGTCATGGTGAGCAAGGGC-3′ and the reverse primer, 5′-CAAACTGCATTACCTGATCCATAGATCTGAGTCCGGACTTG-3′. GFP–Sec61β (Rolls et al., 1999) was obtained from Tom Rapoport (Harvard University, Cambridge, MA), and GFP-Pex26 was obtained from Peter Kim (University of Toronto, Toronto, Canada). To construct *GFP–fs-Sec61b*, a single cytosine was inserted using restriction enzyme-free cloning (van den Ent and Löwe, 2006) with forward primer, 5′-CGATTCTACACAGAAGATTCACCTGG-3′ and reverse primer, 5′-GCTCAAAGCTTGGCCCTGT-3′ using *GFP–Sec61b* as template.

### Cell culture, fractionation, transfection, microinjection, FISH and immunofluorescence

Cell culture, DNA transfection or microinjection, digitonin extraction, FISH staining and immunostaining were performed as previously described (Gueroussov et al., 2010; Cui and Palazzo, 2012; Cui et al., 2012). BAT3-knockout MEF cells were obtained from Hitoshi Okada (University of Toronto, Toronto, Canada) (Sasaki et al., 2007). BAT3^−/−^ cells were grown in DMEM supplemented with 10% FBS and 2-mercaptoethanol. U2OS and COS7 cells were transfected using GenJet Transfection Reagent (SignaGen Laboratories). BAT3^−/−^ and MEFs cells were transfected using JetPrime Polyplus (Invitrogen) transfection reagent. Cell fractionation was performed as previously described (Cui et al., 2013). Samples were separated by SDS-PAGE and analyzed by western blotting using rabbit polyclonal antibodies against Trapα (dilution 1:1000; Görlich et al., 1990), Sec61β (dilution 1:1000; Görlich et al., 1992), GAPDH (dilution 1:1000; Abgen) and GFP (dilution 1:1000; Molecular Probes), and monoclonal mouse antibody against α-tubulin (dilution 1:1000; Sigma). To detect t-ftz protein, which contains an HA epitope (Palazzo et al., 2007), samples were immunoblotted with anti-HA mouse monoclonal antibody (GeneTex; dilution 1:1000).

For FISH staining, the deoxyoligonucleotides used to recognize *ftz* (5′-GTCGAGCCTGCCTTTGTCATCGTCGTCCTTGTAGTCACAACAGCCGGGACAACACCCCAT-3′), *ALPP* (5′-CAGCTTCTTGGCAGCATCCAGGGCCTCGGCTGCCTTTCGGTTCCAGAAG-3′), *GFP* (5′-CTCCATCTTATTGCCCAGGATGTTGCCATCCTCCTTGAAATCGGTGCCGG-3′) were conjugated at the 5′ end with Alexa Fluor 546 or Alexa Fluor 647 (Integrated DNA Technologies). Polyclonal rabbit ATP5A antibody was obtained from Angus McQuibban (Abcam, ab14748). Polyclonal rabbit anti-Trapα antibody was obtained from Tom Rapoport (Görlich et al., 1990). For immunofluorescence staining, permeabilized cells were stained with primary antibodies at 1:200 dilution for Trapα, and 1:2000 for ATP5A for 1 h at room temperature. The secondary goat anti-rabbit-IgG antibody (conjugated to Alexa Fluor 647; Molecular Probes) was used at 1:500 dilution for 30 min at room temperature. All reagents were purchased from Sigma-Aldrich unless otherwise specified.

Fluorescence imaging and FISH quantification were performed as described previously (Gueroussov et al., 2010; Cui et al., 2012; Cui and Palazzo, 2012). All *P*-values were calculated using a Student's unpaired *t*-test.

### Lentiviral-mediated shRNA knockdown

Lentiviral-mediated shRNA knockdown was performed as previously described (Cui et al., 2012). Plasmids encoding shRNA against p180 (clone B9, TRCN0000117407 and clone B10, TRCN0000117408, Sigma), BAT3 (TRCN0000007357, Sigma), TRC40 (clone A, TRCN0000042959 and clone B, TRCN0000042960, Sigma), nesprin-2 (also known as SYNE2; TRCN0000303799, Sigma) or empty vector (pLKO.1) were transfected into the HEK293T cells together with the accessory plasmids, VSVG and Δ8.9, to generate lentivirus carrying specific shRNA plasmids. U2OS cells were infected with lentivirus for 3–4 days and selected using 2 ng/μl puromycin. The level of knockdown was examined using western blotting analysis and was performed as described previously (Cui et al., 2012). Antibodies against BAT3 and TRC40 were obtained from Manu Hegde (Mariappan et al., 2010) and used at 1:1000 dilution.

### Visualization and quantification of endogenous mRNA

The localization of endogenous *Sec61b*, nesprin-2 (*SYNE2*) and *GAPDH* mRNA was visualized using customized or cataloged Stellaris probe arrays (Biosearch Technologies, Petaluma, CA) against human and mouse genes*.* U2OS or MEF cells were grown on coverslips, either treated with control or HHT-containing medium for 30 min, then fixed directly or after digitonin extraction. The staining was performed as per the manufacturer's protocol with the following exception: after overnight staining with FISH probes, the cells were washed three times with 2× SSC solution containing 10% formamide at room temperature. After washing, the cells were mounted and visualized. After cells were imaged using phase microscopy, the number of endogenous mRNA foci in each cell was quantified using NIS Element software (Nikon Corporation, Tokyo, Japan). Briefly, cell and nuclear peripheries were selected to generate regions of interest (ROIs). Then, the number of endogenous mRNA foci was counted using the ‘spot detection’ function, selecting for bright spots that were about 0.32 μm in diameter.

## Supplementary Material

Supplementary information
