# Supplementary material for: mRNA encoding Sec61β, a tail-anchored protein, is localized on the endoplasmic reticulum
Source: J Cell Sci. 2015 Sep 15;128(18):3398–410. doi: 10.1242/jcs.168583 (PMC4582399; doi:10.1242/jcs.168583)
Supplement: Supplementary information [file supp_128_18_3398__index.html]

Supplementary information 

# mRNA encoding Sec61β, a tail-anchored protein, is localized on the endoplasmic reticulum

## JCS168583 Supplementary information

**Files in this Data Supplement:**

- Supplementary information
