# Supplementary material for: mRNA encoding Sec61β, a tail-anchored protein, is localized on the endoplasmic reticulum
Source: J Cell Sci. 2015 Sep 15;128(18):3398–410. doi: 10.1242/jcs.168583 (PMC4582399; doi:10.1242/jcs.168583)
Supplement: Supplementary information [file supp_jcs.168583_JCS168583supp.pdf]

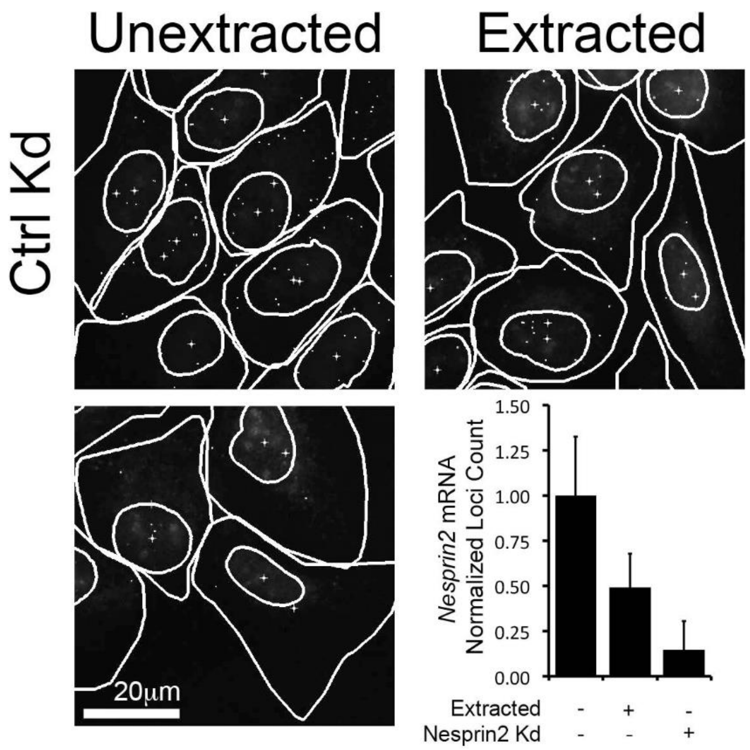

Fig. S1. *Nesprin2* mRNA staining is specific.

U2OS cells were treated with lentivirus containing control ("Ctrl Kd") or anti Nesprin2 shRNAs ("Nesprin2 Kd") for 3 days. Cells were either fixed directly ("Unextracted") or first treated with digitonin ("Extracted"), and stained using FISH probes against endogenous human *Nesprin2* mRNA. mRNA FISH signals overlaid with the contours of the cells and nuclei with detected mRNA foci positions are shown. The percentage of ER-associated foci was determined for each condition by normalizing to the number of foci in the unextracted, control shRNA treated cells. Each bar is the average and standard error of 30 cells. Scale bar = 20μm.

Journal of Cell Science • Supplementary information

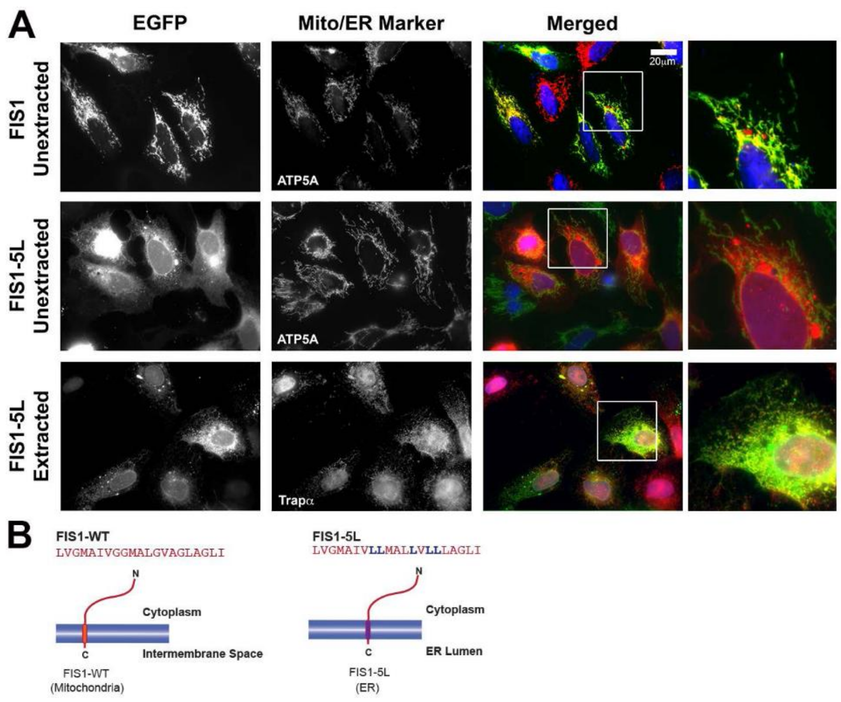

Fig. S2. Incorporation of leucines in the TMD of FIS1 reroutes the protein from the mitochondria to the ER.

(A) COS7 cells were transfected with plasmid encoding GFP-FIS1 or GFP-FIS1-5L, which contains 5 leucine mutations (see the sequence in panel B). Cells were either directly fixed ("unextracted') or first extracted with digitonin then fixed. The fixed cells were stained with DAPI and immuostained for either ATP5A (a mitochondrial marker), or Trapα (an ER marker). Each row represents a single field of view including overlays of GFP-FIS1/FIS1-5L (green), ATP5A/ Trapα (red) and DAPI (blue). Scale bar = 20µm.

Journal of Cell Science • Supplementary information
